# Maintaining the joy of discovery

**DOI:** 10.7554/eLife.80711

**Published:** 2022-06-28

**Authors:** Eve Marder

**Affiliations:** 1 Volen Center and Biology Department, Brandeis University Waltham United States

**Keywords:** living science, graduate school, publishing

## Abstract

Changes in science over the past 50 years have reduced the chances of trainees experiencing the joy of discovery.

My undergraduate honors thesis supervisor, Andrew Szent-Györgyi, was spare with advice, but soon after I started working in his laboratory he said: “The more you put into your work, the more you will get from it”. He didn’t bother to elaborate on that statement, but I understood his message immediately. I believe his message is as relevant today as it was when I was a fledging scientist.

As I watch my own trainees, I am struck by how much more difficult it is for them than it was for me. Fifty years ago, most basic science came from small groups of investigators, working independently, and there was lots of “low-hanging fruit”. Today, research groups are much bigger, papers have more authors, and there is little low-hanging fruit. Fifty years ago, we were in direct contact with the data, and often knew rapidly whether the experiment had worked and what it showed. Even as students and postdocs, many of us were the first in the world to see something, and this joy of discovery – even for something small – fueled our passion for doing science. I fear that the greater technological and statistical complexity of the work done today may partially occlude that joy of discovery.

In my early years, I analyzed each experiment as I did it. This “analysis” might have meant plotting the data and or using a ruler to measure things. Today it can take days or weeks to analyze data, so it is understandable that some researchers prefer to keep collecting data, and wait for its analysis. However, without ready analysis, we deprive ourselves of the immediate satisfaction of seeing the results and, maybe, of knowing that we have indeed seen something for the first time. And if the data are analyzed by someone else – as is becoming more common – we may lose our sense of ownership of the work. Leaving aside the instances, such as in clinical trials, when analysis is purposely detached from execution, my suggestion would be that trainees get in the habit of doing analysis contemporaneously with data collection, so that they are constantly looking at their data.

When I started graduate school, I went to the library and read the tables of contents of the new journals to see what had been published that week/month. As a postdoc in Paris, I had to go to three different libraries to find the journals I routinely followed. Even so, in several hours a week, supplemented by lab conversations, I developed a sense of “what was happening” in the field. Indeed, early in my career I felt I was an “expert” in my field. Today, it is more difficult for a trainee to become oriented in the literature: it may be possible to browse journals without leaving your desk or lab, but the benefits of being able to do this are compromised by the almost infinite size of the literature, and it is harder for any of us to feel we are an “expert”.

I often ask my students and postdocs to do literature searches to determine whether something is known, or if someone has already done an experiment. Sometimes I perform such searches myself, and from time to time I find papers that my trainees did not. I recently asked two of my postdocs about this, and wondered if we were using different search terms and strategies. They shook their heads at my naiveté, smiled sweetly, and explained that Google and other search engines keep track of our search histories and how we access the literature, so they bring different papers to the attention of different people. This made me realize that it would be wise to have several people independently search for relevant literature at various stages of a project.

**Figure 1:**
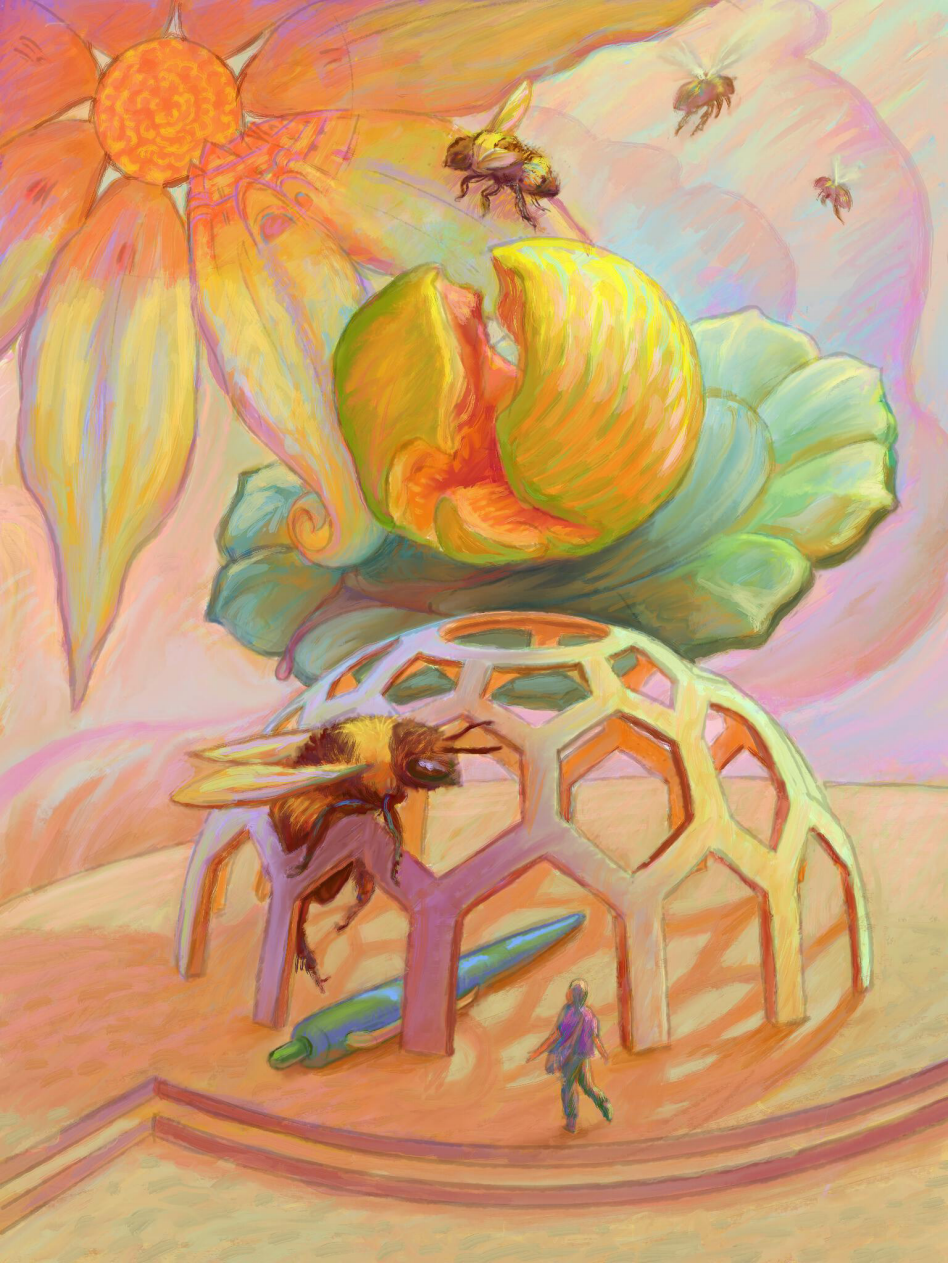
It is important that students and postdocs are involved in writing papers as part of their training.

Illustration: Ben Marder.

Many scientists struggle to write, and for as long as I can remember, I have had colleagues who claimed to hate writing. I always found this somewhat mystifying, because to me, writing the results of a set of experiments is part of the discovery process. Oftentimes, I finally understood the potential significance of a piece of work while writing the paper. In fact, the hardest papers to write are often the most important, precisely because their implications only become clear after multiple iterations. (Likewise, as I write these commentaries for *eLife*, it is often in their evolution that I discover more precisely what I want to say.) It is important, therefore, that students and postdocs are involved in the writing of papers as part of their training.

This year a colleague and I taught a writing course. Most of the students were undergraduate science majors. As we watched the students attempt to find words, sentences and paragraphs that captured what they wanted to say, it dawned on us that many in this cohort had not read or written as much as we had by their age. This is why, several years ago, I started telling trainees to read books – any books – if they wanted to become better writers. My hope is that reading more will give our trainees a better ear for the English language, a better sense of nuance in choosing words, and thus aid them to find the voice they need to communicate their findings.

Working in a large-scale collaboration can present challenges: playing a small role in a much larger project can provide the individual with a sense of moving science towards a lofty goal that could never be achieved by a single person or small laboratory. The downside is that the way that such collaborations write papers can reduce the joy of discovery for the trainees involved, and may reduce their sense of ownership of their own work. On the other hand, many large collaborations, companies and stand-alone institutes do an excellent job of maintaining staff engagement in their projects.

We are all aware of the uncertainties and stresses of finding academic jobs in today’s environment. However, this is partially mitigated by remarkable opportunities in industry, government and publishing. When trainees leave academic science for these opportunities, we should celebrate their success. And regardless of the path they follow, we should hope that our trainees retain their joy in science, and their ability to develop a sense of mastery of their fields.

## Note

This essay is part of the Living Science collection.

